# In Trouble

**Published:** 1893-02

**Authors:** 


### EDITORIAL DEPARTMENT.

F. E. DANIEL, M. D., Editor.
A. J. SMITH. M. D., Galveston, Associate Editor.

EDITORIAL STAFF:

PROF. J. E. THOMPSON, M. D., Texas Medical College, Galveston; Surgery.
PROF. WM. KEILLER, M. D., Texas Medical College, Galveston; Obstetrics and Gynecology.
PROF. DAVID CERNA, M. D., Texas Medical College, Galveston; Therapeutics.
PROF. A. J. SMITH, M. D., Texas Medical College, Galveston; Medicine.
DR. ISADORE DYER, Tulane University; Dermatology.
DR. R. H. L. BIBB, Saltillo, Mexico; Foreign Correspondent.
DR. ROBT. MORRIS, Charity Hospital, N. O.; Clinical Reports.

The JOURNAL is the official organ of the Austin District Medical Society, the West Texas District Medical Society and the Galveston County Medical Society.

### IN TROUBLE.

A little publication called *Printers' Ink* (we have never more than glanced at a copy, but have always understood it to be a kind of an advertising dodge for gratuitous distribution), seems to have been refused passage through the mails as a "journal," i. e., as second class matter, and is loud in its wails, and still louder in its denunciation of Postmaster General Wannamaker. Medical publishers have been besieged lately with communications from *Printer's Ink*, begging them to take sides with it in its fight, and to help get it "legitimatized," it says. The latest publication the JOURNAL has read, is a postal card, whereon is printed a request from a Y. M. C. A. reading room for a free copy, a complaint that Wannamaker will not let it go, and a statement from the Assistant Postmaster General to Rowell & Co., that no legitimate second class matter has ever been refused the mails; from all of which we infer that *Printers' Ink* has been pronounced by the Postoffice Department to be not second class matter, and not entitled to the mails.

That is about all there is in it, that we can see. *Printers' Ink* complains that other matter, intended for advertising or for electioneering, is passed through the mail. Well, what of it? Two, nor three, wrongs would not make a right; besides, it is not *Printers' Ink's* say, whether the publications cited are ligitimate second class matter or not; it is Mr. Wannamaker's say; and it seems he has begged leave to differ with brother *Printer's*

*Ink*, and has said they are. Well, what are you going to do about it, brother P. I.? No thief ever felt the halter draw and had much of an opinion of the law the halter or the draw, or words to that effect, in the language of the late lamented Arte-mus Ward.

If Brother Wannamaker would draw the line a little more closely, and exclude a big lot more of the P. Ink sort, he would be doing the country a service, and it would be but justice to legitimate medical and other "trade" journals.

